# Multi-Environment Evaluation and Stability Analysis for the Selection of Elite Pearl Millet Genotypes with Better Fodder Yield and Quality Component Traits

**DOI:** 10.3390/plants15071034

**Published:** 2026-03-27

**Authors:** Shashikumara Puttamadanayaka, Manjanagouda S. Sannagoudar, Chandra Nayaka Siddaiah, Vinod Kumar, Brijesh Kumar Mehta, Anup Kumar, Krishna Kumar Dwivedi, Govintharaj Ponnaiah, Shashi Kumar Gupta

**Affiliations:** 1 Indian Grassland and Fodder Research Institute, ICAR, Jhansi 284003, India; brijeshmehtaiari@gmail.com (B.K.M.); akanup24@gmail.com (A.K.); dwivedi1976@gmail.com (K.K.D.); 2 ICAR—National Institute of Seed Science and Technology, Regional Station, Bengaluru 560065, India; mssagron@gmail.com; 3 AICRP on Pearl Millet, University of Mysore, Mysore 570005, India; moonnayak@gmail.com; 4 ICAR—Indian Grassland and Fodder Research Institute, Regional Station, Dharwad 580005, India; vinoddhone@yahoo.com; 5 International Crops Research Institute for the Semi-Arid Tropics, Hyderabad 502324, India; govinth.tnau@gmail.com

**Keywords:** pearl millet, fodder yield, lignin, multi-environment trial, AMMI, WAAS, MTSI, genotype × environment interaction

## Abstract

The development of stable and high-yielding fodder pearl millet genotypes with improved quality traits is crucial for enhancing livestock productivity under diverse environments. In this study, twenty-six elite genotypes, including brown midrib (bmr) lines and two check cultivars, were evaluated across four locations, which fall broadly under two agro-climatic zones of India, during the summer season of 2024 to assess their stability for yield and fodder quality traits. Significant genotypic differences and genotype × environment interactions (GEIs) were observed for all traits, indicating substantial genetic variability and environmental influence on trait expression. Additive Main Effects and Multiplicative Interaction (AMMI) and Weighted Average of Absolute Scores (WAAS) analyses identified IGPM 100 as a high-yielding and stable genotype across environments, whereas Baif Bajra 1 and IGBV 97 exhibited specific adaptation. Among quality traits, ICMbmr 2401, ICMbmr 2402, and ICMbmr 2404 recorded consistently low lignin content, confirming their potential for improving forage digestibility. Further, ICFPM 05 recorded high tillering and longer leaves, while ICMFV 2308 exhibited late flowering across locations, indicating their potential for use in developing leafy, late-flowering genotypes. The multi-trait stability index (MTSI) efficiently identified IGPM 100, ICFPM 02, ICMbmr 2404, and IGBV 9 as superior and stable genotypes across multiple traits. High selection differentials for green fodder yield and negative differentials for lignin and fibre fractions highlight the possibility of a simultaneous improvement in yield and quality traits. Overall, the integration of AMMI, WAAS, and MTSI models facilitated the identification of broadly adapted and trait-specific genotypes, which, after evaluating their combining ability, can be used for developing fodder pearl millet composites and hybrids.

## 1. Introduction

Pearl millet (*Pennisetum glaucum* (L.) R. Br.) is a climate-resilient and drought-tolerant major cereal crop, which is widely cultivated for grain and fodder purposes. It is a C4 crop with a better root system, and its ability to tolerate high temperature makes this crop highly suitable for both grain and fodder production. Given these potential resilient traits, this crop is cultivated on approximately 30 million hectares globally and occupies 6.7 million hectares in India [1]. It is a promising fodder crop suitable for rainy and summer seasons and contains on average 7 to 10% crude protein, 56 to 64% NDF, 38 to 41% ADF, 33 to 34% cellulose and 18 to 23% hemicellulose on a dry matter basis, along with minimal anti-nutritional factors like hydrocyanic and oxalic acid [2,3]. Considering its wider adaptability, regrowth potential, and drought and heat tolerance, this crop is popular during the summer season while providing continuous green fodder through three to six cuts per season [4].

Fodder yield in pearl millet is associated with traits like days to 50% flowering, plant height, high tillering ability, regrowth potential and the leaf-to-stem ratio. Similarly, fodder quality considers acid detergent fibre, neutral detergent fibre, lignin (%), crude protein and in vitro organic matter digestibility. The primary aim is to develop high-biomass cultivars with better fodder quality. Hence, developing trait-specific genotypes for key fodder yield and quality traits and conducting evaluations in multilocation trials help in identifying superior stable genotypes and specific genotype responses for targeted areas with unique traits [5]. Such trait-specific genotypes can be used as donor lines in breeding programs for developing superior cultivars.

Among the different stability analysis methods involved in the evaluation of multilocation data, the AMMI model and GGE biplots are considered highly effective for easily assessing genotype stability, as well as the combination of stability and yield performance across different environments [6,7]. Previous studies analyzed genotypic × environment interactions using AMMI and GGE biplot analysis, including a study by [8], who identified stable, high-yielding finger millet genotypes across different agroecological zones of India. Similarly, stability analysis was employed for selecting high-iron and -zinc genotypes in pearl millet [9]; high-yielding genotypes in oat [10] and high-yielding sorghum genotypes for the humid lowlands of Ethiopia [11]. These studies analyze GEIs and select stable genotypes for use in breeding programs.

Fodder yield is influenced by several key traits, including plant height, days to 50% flowering, leaf-to-stem ratio, total number of tillers, leaf length and width, regrowth potential, and overall biomass production. The summer season (March to June) is typically considered a lean period, during which a significant scarcity of green fodder is observed. This shortage often leads to reduced milk production and overall livestock productivity. In recent years, fodder pearl millet has gained popularity in India as a valuable summer fodder crop. It is also cultivated as summer pasture in regions such as the southern United States, Brazil, and Central Asian countries [12,13]. Forage digestibility is closely linked to the quality of biomass and plays a critical role in determining milk and meat productivity in livestock. One of the primary limiting factors for digestibility is lignin, a complex and indigestible phenolic polymer. Lignin impedes the access of cellulolytic enzymes and rumen microorganisms to structural carbohydrates such as cellulose and hemicellulose. Consequently, lignin content is negatively correlated with both in vitro and in vivo cell wall digestibility. Reducing lignin levels directly enhances dry matter digestibility and also improves the efficiency of bioethanol production. The brown midrib (bmr) lines with characteristic reddish-brown to tan-coloured midribs in leaf blades contrast with the pale green midrib leaf blades associated with reduced lignin content and altered lignin composition, traits very useful for improving forage digestibility for livestock and bioethanol production [14,15]. Our aim is to identify stable germplasm lines suitable in the summer season for different fodder yield component traits and identify stable, low-lignin genotypes.

The multi-trait stability index (MTSI) is useful in selecting high-yield and stable genotypes in METs based on multiple traits considering both fixed and random effects models [6]. This index enables the selection of stable genotypes by identifying those with a positive selection differential for traits targeted for improvement and a negative differential for traits targeted for reduction. Moreover, it serves as a valuable tool for breeders aiming to select genotypes based on both average performance and stability across multiple traits. The index offers a simplified and interpretable selection process while accounting for the correlation structure among traits.

We developed unique, elite trait-specific fodder pearl millet lines, as this material is associated with multicut fodder yield, higher leaf biomass and brown midrib (bmr) lines, which are the least exploited in pearl millet for developing fodder pearl millet lines with higher digestibility. Hence, in this study, a total of 26 unique pearl millet genotypes were evaluated across different agroecological zones with the aim of determining potential genotypes, the stable expression of traits, GEI effects in trait expression and their adaptability in a wide range of environments.

## 2. Results

### 2.1. Additive Main Effects and Multiplicative Interaction (AMMI)

A combined analysis of variance (ANOVA) was performed for 12 traits across 26 genotypes evaluated in four environments. Highly significant (*p* < 0.01) differences among genotypes were observed for all traits (Table 1). This indicates sufficient genetic variability in the population for fodder yield traits (GFYFC, GFYSC, GFYTC, PH, TNT), leaf dimensions (LL, LW, LS ratio) and quality traits (ADF, NDF, lignin). Environments differed significantly for most traits except lignin, indicating the importance of environmental conditions in trait expression. Replication effects were mostly non-significant, with the exception of a few traits, TNT, LS ratio and GFYTC, where small but significant effects were detected. Residuals contributed little to the total variation in most traits except for DFF, PH and LL, confirming good experimental precision.

**Figure 1:**
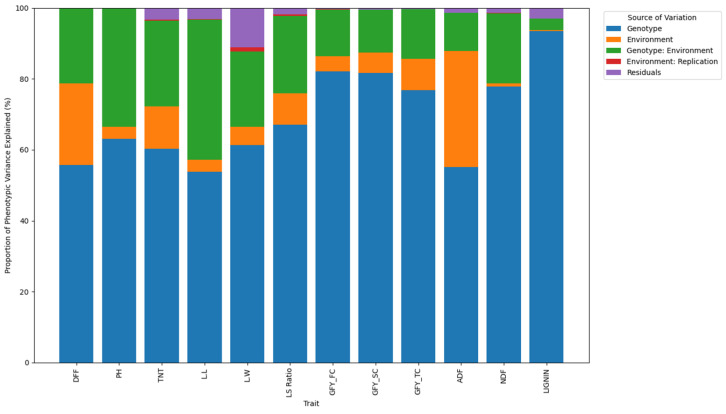
Proportion of phenotypic variance for studied traits in fodder pearl millet genotypes. DFF: days to 50% flowering; LL: leaf length; LW: leaf width; TNT: total number of tillers; PH: plant height; LS Ratio: leaf-to-stem ratio; ADF: acid detergent fibre; NDF: neutral detergent fibre; GFYFC: green forage yield at first cut; GFYSC: green forage yield at second cut; GFYTC: green forage yield at third cut.

The partitioning of phenotypic variance using the AMMI model (Table 2; Figure 1) revealed that genotypic effects were the predominant source of variation across most fodder yield and quality traits in pearl millet. The proportion of phenotypic variance attributed to genotype ranged from 53.8% (LL) to 93.4% (lignin), indicating strong genetic control and suggesting ample scope for selection-based improvement. Genotypic variance contributed more than 75% of the total variation for GFYFC, GFYSC, GFYTC, NDF, and lignin, highlighting their relative stability and heritable nature. In contrast, environmental effects explained a smaller fraction of the total variance, being comparatively higher for DFF (23.1%) and ADF (19.5%), suggesting the sensitivity of these traits to environmental fluctuations. The genotype × environment interaction (GEI) contributed moderately to several traits such as PH (33.4%), LL (39.4%), and TNT (24.2%), reflecting variable genotypic performance across testing locations. The effects of replication and residual error were minimal (<5%), confirming the high experimental precision and reliability of the phenotypic evaluations.

Table 2 summarizes the four location averages for the measured traits. The highest days to flowering (DFF) value was observed in ICMFV 2308 (117.25 days), while plant height (PH) was the greatest in IGBV 128 (175.99 cm). Total tillers per plant (TNT, 16.05) and leaf length (LL, 78.80 cm) were the highest in ICFPM 05; leaf width (LW) peaked in ICFPM 06 (3.69 cm); and the leaf:stem ratio (LSR) was the highest in IGPM 100 (2.01). Green fodder yields at the first and second cuts were the highest in Baif bajra 1 (20.06 kg/plot and 13.97 kg/plot, respectively), whereas GFYTC (9.25 kg/plot) was the highest in IGBV 97. Among fodder quality traits, lignin (%) is considered undesirable because lower lignin content, along with lower ADF and NDF, improves digestibility; the lowest mean lignin content (3.06%) was recorded in ICMbmr 2402. The lowest ADF values were observed in ICMbmr 2401 and ICMbmr 2402 (29.61% and 29.77%, respectively), and the lowest NDF content was recorded in IGPM 100 (54.06%).

**Figure 2:**
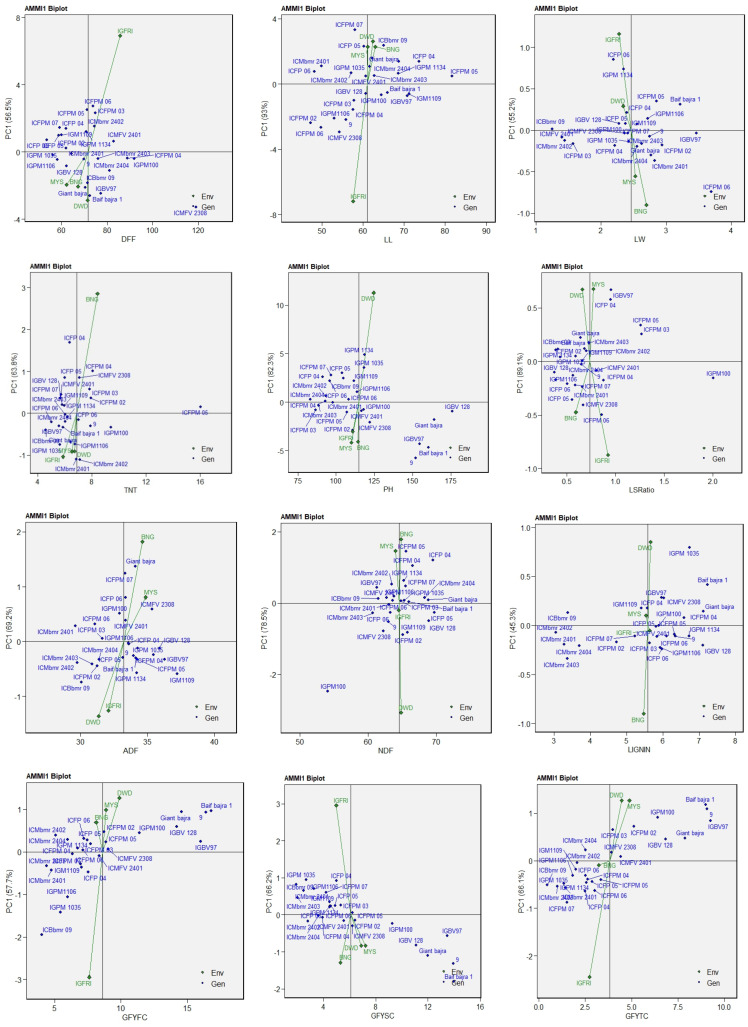
AMMI biplot for different fodder yield and quality component traits showing interaction of IPCA2 against IPCA1 scores of 26 fodder pearl millet genotypes (G) in four environments. DFF: days to 50% flowering; LL: leaf length; LW: leaf width; TNT: total number of tillers; PH: plant height; LS Ratio: leaf-to-stem ratio; ADF: acid detergent fibre; NDF: neutral detergent fibre; GFYFC: green forage yield at first cut; GFYSC: green forage yield at second cut; GFYTC: green forage yield at third cut.

The AMMI1 analysis revealed substantial genotype × environment interactions (GEIs) across morpho-physiological and fodder quality traits in pearl millet. For days to 50% flowering (DFF), the first principal component (PC1) accounted for 56.5% of the GEI (Figure 2), with genotypes ICMbmr 2401, IGPM 100, and ICMbmr 2404 showing high stability across environments, whereas ICFPM 06, ICFPM 03, and ICMbmr 2402 were specifically adapted to the IGFRI environment. ICMFV 2308 was identified as the latest-flowering genotype. For the total tiller number (TNT), ICFPM 05 exhibited the highest and most stable tillering ability. As for the leaf:stem ratio (LSR), PC1 explained 89.1% of the interaction, with IGPM 100 combining a high mean LSR and low interaction score, indicating superior stability and leafiness across locations. The AMMI1 biplots for leaf length (LL), leaf width (LW), and plant height (PH) explained 93.9%, 55.2%, and 82.3% of the GEI variation, respectively. Genotypes ICMbmr 2401, ICFPM 03, and ICFPM 04 were identified as broadly adapted and stable, while IGBV 128, Giant bajra, and Baif bajra 1 exhibited specific adaptation to the DWD and BNG environments. For green fodder yield, the first and second cuts (GFYFC and GFYSC) accounted for 57.7% and 66.2% of the GEI variation, respectively. Genotypes ICMbmr 2401, ICFPM 03, and IGPM 1035 showed high stability and broad adaptability, whereas IGBV 97, Giant bajra, and Baif bajra 1 were specifically adapted to BNG and MYS, the most discriminating environments. At the third cut (GFYTC), Baif bajra 1 and IGBV 97 exhibited high mean yields but specific adaptation to MYS and DWD, while IGPM 100 showed high yield and moderate interaction, suggesting wider adaptability. For fodder quality traits, the AMMI1 biplots explained 69.2% (ADF), 78.5% (NDF), and 45.3% (lignin) of the GEI variation. Genotypes ICMbmr 2401, ICFPM 03, and IGPM 1035 were the most stable and broadly adapted, while Giant bajra, Baif bajra 1, and IGPM 100 showed specific adaptation to BNG and DWD, environments with the strongest discriminatory influence for quality traits.

**Figure 3:**
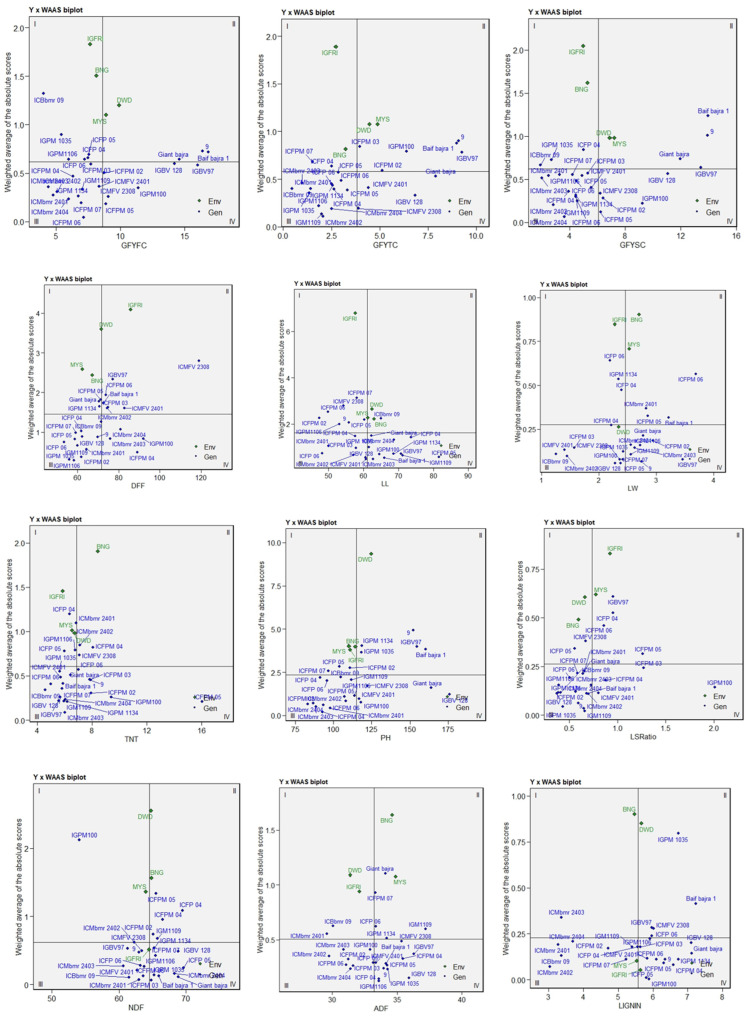
Mean performance vs. WAAS biplot from AMMI model. DFF: days to 50% flowering; LL: leaf length; LW: leaf width; TNT: total number of tillers; PH: plant height; LS Ratio: leaf-to-stem ratio; ADF: acid detergent fibre; NDF: neutral detergent fibre; GFYFC: green forage yield at first cut; GFYSC: green forage yield at second cut; GFYTC: green forage yield at third cut.

The mean performance versus WAAS biplot (Figure 3), unlike the AMMI1 model that relies on the first interaction principal component, estimates stability by integrating all IPC scores, thereby capturing the total variance in the G × E interaction and providing a more comprehensive assessment of genotype stability. The Y × WAAS plot for days to 50% flowering (DFF) classified genotypes into four quadrants based on the mean performance and stability. Genotypes located in quadrant IV, such as IGPM 100, ICMbmr 2401, and ICMbmr 2404, combined early flowering with low WAAS values, reflecting both earliness and stability, whereas ICMFV 2308 in quadrant II was the latest-flowering and least stable genotype. For the total tiller number (TNT), ICFPM 05 and IGPM 100 exhibited high mean values with low WAAS, indicating consistently superior and stable tillering ability. Similarly, IGPM 100 and ICMbmr 2404 recorded longer leaves with moderate WAAS, reflecting wide adaptability for leaf traits. In the WAAS biplot for the leaf:stem ratio (LSR), IGPM 100 ranked the highest for the mean LSR with a low WAAS, identifying it as a highly stable and desirable genotype, while ICMbmr 2401, ICMbmr 2404, and Baif Bajra 1 clustered near the origin, showing moderate means and stability. For green fodder yield at the third cut (GFY_TC), Baif Bajra 1 and IGBV 97 produced the highest mean yields but larger WAAS values, indicating specific adaptation to the Mysore and Dharwad environments.

In contrast, IGPM 100 combined high yield with moderate WAAS, signifying broader adaptability and stability, while ICMbmr 2402 and ICMbmr 2404 displayed lower yields but greater stability. Regarding lignin content, where lower values are desirable, ICMbmr 2401, ICMbmr 2402, and ICMbmr 2404 exhibited the lowest mean lignin with low WAAS, indicating superior forage quality and stability across environments. Conversely, Baif Bajra 1 and IGBV 97 showed higher lignin content and higher WAAS, confirming environment-specific responses. Overall, IGPM 100 emerged as a highly adaptable and stable genotype combining high productivity with balanced forage quality, whereas the ICMbmr lines contributed significantly to quality improvement through consistently reduced lignin content.

### 2.2. Multi-Trait Stability Index (MTSI)

**Figure 4:**
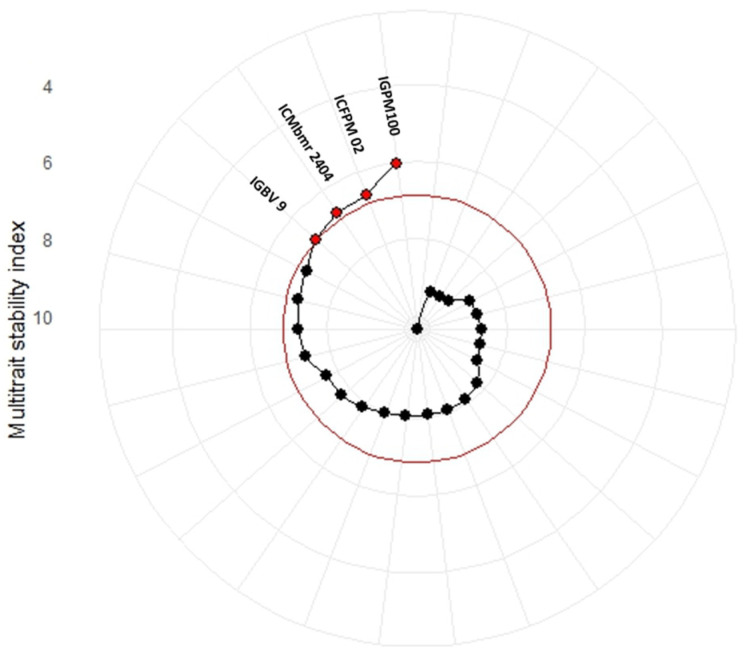
Ranking of fodder pearl millet genotypes in ascending order based on MTSI. (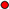 “Selected” and 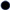 “Non-selected”).

The radial plot of the multi-trait stability index (MTSI) identified genotypes with superior stability across traits. Genotypes closer to the centre had lower MTSI values, indicating greater stability. IGPM 100, ICFPM 02, ICMbmr 2404, and IGBV 9 were selected as the most stable genotypes, while others showed relatively higher MTSI values and were not selected. This highlights the efficiency of the MTSI in multi-trait selection (Figure 4). However, it is important to note that the MTSI reflects predefined selection criteria (trait weighting and direction of improvement) and should therefore be interpreted as a structured selection tool rather than absolute biological evidence of superiority. Significant variation was observed in selection response among the traits studied in the selected genotypes (Table 3). Total fodder yield (GFYTC) and second cut yield (GFYSC) showed the highest selection differentials, followed by first cut yield (GFYFC), indicating strong potential for biomass improvement. Morphological traits such as leaf width and the leaf-to-stem ratio recorded moderate selection response with high heritability, supporting their use as secondary selection criteria. In contrast, plant height and leaf length showed limited improvement scope. For quality traits, ADF, NDF, and lignin showed negative selection differentials with high heritability, particularly lignin (−7.53%), highlighting scope for enhancing digestibility. Overall, yield and quality traits offer the greatest potential for simultaneous genetic improvement.

## 3. Discussion

The selection of trait-specific stable genotypes and multi-trait stable genotypes will be helpful in breeding programs for developing high-yielding genotypes with wider adaptability. Hence, in this experiment, the multilocation evaluation of fodder pearl millet genotypes particularly for traits associated with fodder yield and lignin component traits showed significant genotypic differences for all traits, indicating substantial genetic variability among the evaluated pearl millet genotypes. Significant environmental effects for most traits, except lignin, underline the strong influence of growing conditions on trait expression, emphasizing the need for multi-environment testing. The four experimental sites exhibited significant variations in altitude, rainfall patterns, temperature ranges, and soil properties. Specifically, site E1 (Jhansi), situated within the Central Plateau and Hills Region, is characterized by a broad temperature range of 15–45 °C and sandy loam soil. In contrast, sites E3 (Bangalore), E4 (Mysore), and E2 (Dharwad), located in the Southern Plateau and Hills Region, experience more moderate temperature ranges between 16 and 39 °C. Additionally, E3 and E4 are typified by red sandy loam soils, whereas E2 features red with medium black soils, thereby presenting a spectrum of diverse soil conditions (Table 4).The non-significant replication effects and low residual variance confirm high experimental precision. Similar observations have been reported in wheat [16], sugar beet [17], pearl millet [18], and sorghum [19], supporting the importance of genotype and environment evaluation for identifying stable, high-yielding fodder cultivars.

Variance component analysis revealed that genotypic effects were the predominant source of variation for most traits, indicating strong genetic control and lesser environmental influence. High proportions of genotypic variance for DFF, PH, LL, GFY across cuts, ADF, NDF, and lignin suggest good prospects for selection efficiency and genetic improvement. Among quality traits, bmr lines (ICMbmr 2401, ICMbmr 2403, ICMbmr 2404) recorded low and stable lignin content across sites, confirming their potential for quality improvement. The studies conducted by Cherney et al. [20], on brown midrib (bmr) pearl millet and Hanna et al., [21], on bmr sorghum demonstrated that the reduced lignin content in bmr varieties significantly enhances in vitro organic matter digestibility (IVDMD) compared to conventional pearl millet and sorghum. The presence of bmr gene(s) holds considerable potential for improving fodder digestibility, which consequently leads to increased milk production and enhanced animal performance. The relatively low environmental sensitivity of lignin content is related to the genetically regulated nature of lignin biosynthesis. The brown midrib (bmr) trait is governed by recessive mutations [14,15,22] affecting key enzymes in the monolignol pathway, leading to reduced lignin deposition in cell walls. In sorghum, the bmr6 gene is linked to a decrease in cinnamyl alcohol dehydrogenase (CAD) activity, and the bmr-12 and bmr-18 genes decrease caffeic acid O-methyl transferase (COMT) activity, leading to the modification of monolignol biosynthesis, resulting in a brownish, reddish colour on the leaf midrib and stem [23]. Lignin accumulation in secondary cell walls generally demonstrates reduced phenotypic plasticity relative to growth-related traits. Furthermore, this trait is governed by monogenic control, which accounts for the consistent expression observed in the brown midrib (bmr) phenotype. In contrast, greater environmental effects observed for leaf width and the leaf–stem ratio indicate their higher sensitivity to growing conditions. Significant genotype × environment interactions for LL, LS ratio, GFY_TC, and ADF highlight the necessity of multi-environment evaluation to identify stable and adaptable genotypes, consistent with earlier findings in pearl millet [24]. The combined analysis across four environments indicated significant genotype × environment interactions for most traits (Table 2), confirming the differential response of genotypes under varied conditions. The superior performance of Baif Bajra 1, IGBV 97, and IGBV 128 for fodder yield traits, and ICMbmr 2402 and IGPM 100 for quality traits, suggests the stable and complementary expression of productivity and digestibility traits. The consistency of bmr lines for low lignin content underlines their potential use in improving forage quality. Overall, the presence of wide genetic variability and significant G × E interactions highlight ample scope for selecting stable, high-yielding, and nutritionally superior pearl millet genotypes for multicut fodder systems.

The AMMI1 and WAAS biplot analyses for days to 50% flowering indicated that ICMbmr 2401, IGPM 100, and ICMbmr 2404 exhibited high stability across environments, whereas ICMFV 2308 was the latest-flowering genotype. Furthermore, both the WAAS biplot and AMMI1 analyses identified IGPM 100 as a superior genotype, combining high green fodder yield, greater leafiness, and consistent stability across diverse environments. Both analyses consistently indicated that Baif Bajra 1 and IGBV 97 exhibited specific adaptation, while ICFPM 05 maintained stable and high tillering capacity across locations, thereby contributing substantially to overall biomass accumulation. The wide adaptation observed for IGPM 100 may be attributed to its physiological resilience. This genotype has higher leafy biomass, good tillering ability and early ground covering. These traits cause less evapotranspiration, cooler canopy and higher water use efficiency, making this genotype more adaptable to the summer season across locations [25]. IGPM100 can be further evaluated for physiological traits to understand its mechanism of adaptability. Overall, the AMMI analysis identified genotypes with both broad and specific adaptations suitable for diverse production environments, aligning with earlier reports on the utility of AMMI models in dissecting G × E interactions in pearl millet and other forage crops [26,27,28]. Similarly, the WAAS approach effectively discriminated genotypes with broad and specific adaptation patterns, facilitating the simultaneous selection of productive and stable lines across diverse production environments, as also reported in other forage and cereal crops [6,29].

Olivoto et al., [30], reported that the MTSI is a valuable tool for plant breeders to identify superior genotypes for multiple traits using multi-environment data. The MTSI analysis effectively identified genotypes with superior stability across multiple traits. IGPM 100, ICFPM 02, ICMbmr 2404, and IGBV 9 showed the lowest MTSI values, indicating consistent performance and suitability for broad adaptation. Behera et al., 2024 [31], used the multi-trait stability index for selecting stable forage sorghum genotypes and used them in breeding programs for improving genetic gain across different environments. High selection differentials for GFY_TC, GFY_SC, and GFY_FC suggest strong potential for biomass improvement, while leaf width and the leaf:stem ratio also contribute as reliable secondary selection traits. Negative selection differentials for ADF, NDF, and lignin, especially lignin (−7.53%), highlight prospects for enhancing fodder digestibility. Overall, the MTSI proved efficient for a simultaneous improvement in forage yield and quality traits across environments in pearl millet.

## 4. Materials and Methods

Plant materials: The present study consisted of twenty-six selected fodder pearl millet genotypes, including two checks (Supplementary Table S1). These twenty-six genotypes consist of 18 inbreds and 8 open-pollinated varieties (OPVs). These were evaluated at four locations—ICAR-Indian Grassland and Fodder Research Institute, Jhansi (Jhansi); ICAR-Indian Grassland and Fodder Research Institute, Regional station Dharwad (Dharwad); ICAR-Indian institute of seed science and technology, Regional station Bangalore (Bangalore); and University of Mysore, AICRP on Pearl millet (Mysore)—during the summer season of 2024. The crop was planted in the first fortnight of February in a randomized complete block design with two replications. All the recommended packages of practices were applied to obtain the optimum crop stand. Irrigation was provided at an 8–10-day interval after sowing to maintain optimum moisture in the field. The characteristics of the environments are presented in Table 1. The experimental plots used in this study had two rows, each of which was 3 m long with a 0.40 m interrow spacing.

Observations recorded: The data on different fodder yield and quality traits were recorded in terms of days to 50% flowering. The genotypes were evaluated for plant height (cm), total number of tillers, leaf-to-stem ratio, leaf length (cm), leaf width (cm) and green forage yield (GFY)/plot (kg), measured following first cut (50 days after sowing), second cut (30 days after first cut) and third cut (30 days after second cut) cutting intervals.

Fodder quality traits: Neutral detergent fibre (NDF), acid detergent fibre (ADF), and lignin (ADL) were analyzed using a sequential procedure as modified by Van Soest et al. [32]. Lignin (ADL) was estimated by treating the residue obtained after ADF determination with 72% H_2_SO_4_, followed by ashing in a muffle furnace at Division of Plant Animal Relationship at ICAR-IGFRI, Jhansi.

Statistical analysis: All statistical analyses were conducted using R version 4.4.3. This work was performed on a cloud-based RStudio environment running Ubuntu 20.04.6 LTS. Analyses were carried out using the agricolae package for AMMI analysis and the metan package for the WAAS plot and MTSI analysis. The MTSI was computed to calculate the mean performance and simultaneous stability in terms of DFF, PH, TNT, LL, LW, LS ratio, GFYFC, GFYSC, GFYTC, ADF, NDF and LIGNIN based on the following equation:

MTSIi = [ Σ(j = 1 to f) (γij − γ_j)^2^ ]^0.5

where MTSIi is the multi-trait stability index of the genotype i, γ ij is the score of the genotype i in the factor j, and γij is the score of the ideal genotype in the factor j. Scores were calculated based on factor analysis for genotypes and traits.

The selection differential for each individual trait was then calculated as the difference between the mean of the selected genotypes and the overall population mean for that trait. It was expressed both in absolute terms (SD = ${\overset{-}{X}}_{s}-{\overset{-}{X}}_{o}$) and as a percentage relative to the population mean $\left[SD(\%)=(({\overset{-}{X}}_{s}-{\overset{-}{X}}_{o})/{\overset{-}{X}}_{o})\times 100\right]$.

## 5. Conclusions

The multi-environment evaluation successfully discerned pearl millet genotypes exhibiting both stability and superior performance in terms of fodder yield and quality traits. The integrated application of the AMMI, WAAS, and MTSI models demonstrated efficacy in the concurrent selection of genotypes that are productive, nutritionally enhanced, adaptable to diverse ecological conditions, and stable across multiple locations. These trait-specific fodder pearl millet lines provide valuable genetic resources for use as parents in breeding programs aimed at improving green fodder yield, leafiness, and reduced lignin content in pearl millet, particularly for summer season adaptation.

## Figures and Tables

**Table 1:** Additive effects analysis of variance of AMMI model for studied traits of pearl millet genotypes.

Source of Variation	df	Sum of Squares											
		DFF	PH	TNT	LL	LW	LS Ratio	GFYFC	GFYSC	GFYTC	ADF	NDF	LIGNIN
Genotype	25	38,251.08 **	133,035.27 **	929.53 **	13,941.11 **	68.09 **	24.73 **	2962.73 **	2818.24 **	1401.56 **	818.71 **	2201.05 **	313.36 **
Environment	3	15,846.23 *	6962.65 **	184.36 **	894.41 *	5.62	3.25 **	151.69 **	194.69 **	161.53 **	485.77 **	23.12 **	1.22 **
Genotype: Environment	75	14,633.77 *	70,396.26 **	372.95 *	10,210.02 **	23.67 **	8.05 **	476.48 **	419.72 **	256.15 **	157.94 **	561.00 **	10.62
Environment: Replication	4	0.08	74.81	6.51 *	51.06	1.20	0.12 *	2.46	0.20	0.83*	1.02	1.16	0.08
Residuals	100	15.92 **	298.54 *	50.30	825.78 **	12.43	0.70	14.88	17.32	6.02	20.41	41.22	10.10

DFF: Days to 50% flowering; PH: Plant height; TNT: Total number of tillers; LL: Leaf length; LW: Leaf width; LS Ratio: Leaf-to-stem ratio; GFY_FC: Green forage yield at first cut; GFY_SC: Green forage yield at second cut; GFY_TC: Green forage yield at third cut. * significance at 5% level and ** significance at 1% level.

**Table 2:** Mean data of four locations for different fodder yield and quality component traits in elite pearl millet genotypes.

	G. Name	G. No	DFF	PH	TNT	LL	LW	LS Ratio	GFYFC	GFYSC	GFYTC	ADF	NDF	LIGNIN
1	ICMbmr 2401	IGPM-1	64.50	97.75	8.13	48.01	2.82	0.66	10.02	4.17	2.38	29.61	63.03	3.28
2	ICMbmr 2402	IGPM-2	74.38	93.36	10.24	56.11	1.44	0.70	9.65	4.72	3.45	29.77	63.45	3.06
3	ICMbmr 2403	IGPM-3	76.00	88.67	8.10	61.68	2.62	0.73	9.07	3.38	1.65	30.89	60.69	3.37
4	ICMbmr 2404	IGPM-4	81.00	83.13	7.62	66.63	2.55	0.56	11.72	6.05	4.17	31.40	68.29	3.70
5	ICFPM 02	IGPM-6	62.00	110.85	10.31	49.59	2.94	0.59	17.27	10.72	8.55	31.26	65.11	4.71
6	ICFPM 03	IGPM-7	74.88	86.66	9.84	58.89	1.57	1.27	15.52	8.90	6.63	31.63	64.83	5.63
7	ICFPM 04	IGPM-8	89.00	92.85	11.00	59.02	2.21	0.88	12.68	6.57	4.40	35.47	66.53	6.59
8	ICFPM 05	IGPM-9	71.25	106.12	16.05	78.80	2.85	1.26	17.73	10.40	5.57	34.24	65.54	6.31
9	ICFPM 06	IGPM-10	73.75	108.09	7.50	52.59	3.69	0.86	14.42	7.63	5.02	31.09	63.62	6.10
10	ICFP 04	IGPM-11	62.00	91.27	7.32	73.67	2.40	0.95	15.98	8.33	4.17	33.61	69.50	5.56
11	ICFP 05	IGPM-12	58.75	103.80	8.31	56.89	2.38	0.55	14.93	8.15	4.77	33.15	69.68	5.81
12	ICFP 06	IGPM-13	53.50	95.80	7.53	46.95	2.19	0.50	14.32	7.52	4.17	33.36	63.24	5.97
13	ICFPM 07	IGPM-14	59.25	96.71	8.47	54.26	2.41	0.58	14.43	7.03	2.52	33.32	65.47	5.23
14	ICMFV 2308	IGPM-15	117.75	121.11	8.28	56.98	2.36	0.67	18.32	10.37	6.52	35.34	62.27	6.02
15	ICMFV 2401	IGPM-16	82.88	116.64	6.57	59.13	1.40	0.80	17.00	9.28	7.35	33.37	62.70	5.83
16	ICBbmr 09	IGPM-17	71.50	104.75	6.31	61.80	1.25	0.41	10.33	3.22	0.75	30.08	61.52	3.37
17	IGPM100	IGPM-18	92.00	117.26	12.64	66.36	2.36	2.01	22.83	15.43	10.67	32.93	54.06	5.89
18	IGBV97	IGPM-19	77.25	154.67	7.41	71.11	3.46	0.95	32.55	22.45	15.42	36.29	61.27	5.97
19	IGBV 9	IGPM-21	69.75	152.20	9.57	58.13	2.74	0.65	32.60	21.45	15.12	33.96	63.01	6.35
20	Baif bajra 1	IGPM-22	72.50	160.37	6.01	67.44	3.21	0.68	33.43	23.28	14.98	34.14	65.97	7.35
21	Giant bajra	IGPM-23	70.50	164.04	8.87	59.63	2.77	0.73	28.77	19.97	13.15	34.11	68.85	7.12
22	IGBV 128	IGPM-24	62.25	175.99	7.26	60.91	2.28	0.37	28.37	18.47	11.38	35.91	68.87	7.10
23	IGPM 1134	IGPM-25	70.75	118.50	8.68	69.39	2.34	0.39	13.55	7.47	4.25	34.20	65.26	6.73
24	IGPM 1035	IGPM-26	56.13	118.24	7.44	59.79	2.42	0.43	12.65	4.50	2.23	34.28	65.29	6.74
25	IGPM1106	IGPM-27	58.25	113.65	10.38	55.14	2.72	0.39	13.30	5.47	3.05	33.59	63.79	5.92
26	IGPM1109	IGPM-28	60.00	111.65	7.27	70.71	2.55	0.66	16.50	7.47	3.30	37.21	65.76	5.41

DFF: Days to 50% flowering; PH: Plant height (cm); TNT: Total number of tillers; LL: Leaf length (cm); LW: Leaf width (cm); LS Ratio: Leaf-to-stem ratio; GFYFC: Green forage yield at first cut (t/ha); GFYSC: Green forage yield at second cut (t/ha); GFYTC: Green forage yield at third cut (t/ha); ADF: Acid detergent fibre (%); NDF: Neutral detergent fibre (%); LIGNIN (%).

**Table 3:** Prediction of selection differential for studied traits based on MTSI.

Sr No.	Trait	Xo	Xs	SD	SD Percent	h^2^	Goal
1	PH	115.04	116.22	1.18	1.03	0.824	increase
2	LW	2.46	2.65	0.19	7.71	0.884	increase
3	GFY_FC	8.62	10.6	2.01	24.13	0.946	increase
4	GFY_SC	6.10	8.31	2.21	36.23	0.950	increase
5	GFY_TC	3.82	5.77	1.95	51.05	0.939	increase
6	DFF	71.7	76.2	4.53	6.28	0.872	increase
7	LS Ratio	0.74	0.95	0.21	28.38	0.891	increase
8	TNT	6.87	7.81	0.94	13.68	0.866	increase
9	LL	59.1	61.2	2.1	3.55	0.756	increase
10	ADF	33.2	32.4	−0.85	−2.41	0.936	decrease
11	NDF	64.5	62.6	−1.91	−2.95	0.915	decrease
12	LIGNIN	5.58	5.16	−0.42	−7.53	0.989	decrease

Xo: original value; Xs: selected value; SD: selection differential; SD percent: selection differential in percentage; h^2^: broad-sense heritability. DFF: days to 50% flowering; PH: plant height; TNT: total number of tillers; LL: leaf length; LW: leaf width; LS ratio: leaf-to-stem ratio; GFY_FC: green forage yield at first cut; GFY_SC: green forage yield at second cut; GFY_TC: green forage yield at third cut.

**Table 4:** Geographical characteristics of experimental environments.

S. No.	Environmental Code	Year	Location of Research Station	Altitude (m)	Latitude	Longitude	Rainfall (mm)	Temperature (°C)	Soil Type
1	E1	2024	ICAR-IGFRI, Jhansi, Uttar Pradesh, India	285	25,051′ N	78,053′ E	05.2	15–45	Sandy loam
2	E2	2024	ICAR-IGFRI, Regional station, Dharwad, Karnataka	750	15,048′ N	74,097′ E	115.5	17–38	Red with medium black
3	E3	2024	ICAR-NISST, Bangalore, Karnataka	900	13,008′ N	77,057′ E	181.2	16–38	Red sandy loam
4	E4	2024	University of Mysore, Karnataka	770	12,030′ N	76,063′ E	213.5	16–39	Red sandy loam soil

Temperature and rainfall duration is April to May 2024.

## Data Availability

The original contributions presented in this study are included in the article/Supplementary Materials. Further inquiries can be directed to the first author.

## Supplementary Materials

The following supporting information can be downloaded at: https://www.mdpi.com/article/10.3390/plants15071034/s1, Table S1: Pedigree of Pearl millet genotypes used in this experiment.

## Abbreviations

The following abbreviations are used in this manuscript:

GEIs	Genotype × Environment Interactions
AMMI	Additive Main Effects and Multiplicative Interaction
WAAS	Weighted Average of Absolute Scores
MLT	Multilocation Trial
GGE	Genotype Main Effect Plus Genotype–Environment Interaction
ANOVA	Analysis of Variance
PCs	Principal Components
MTSI	Multi-Trait Stability Index

## References

[1] Satyavathi C.T., Ambawat S., Khandelwal V., Srivastav R.K. (2021). Pearl millet: A climate-resilient nutricereal for mitigating hidden hunger and providing nutritional security. Front. Plant Sci..

[2] Kaushal P., Roy A.K., Malaviya D.R., Bhardwaj N.R., Agrawal R.K., Tonapi V.A., Nepolean T., Gupta S.K., Gangashetty P.I., Yadav O.P. (2024). Forage pearl millet: Issues and strategies for genetic improvement. Pearl Millet in the 21st Century: Food-Nutrition-Climate Resilience-Improved Livelihoods.

[3] Gupta V.P. (1975). Fodder improvement in *Pennisetum*. Forage Res..

[4] Gupta S.K., Govintharaj P., Bhardwaj R. (2022). Three-way top-cross hybrids to enhance production of forage with improved quality in pearl millet (*Pennisetum glaucum* (L.) R. Br.). Agriculture.

[5] Annicchiarico P. (2002). Genotype × Environment Interactions: Challenges and Opportunities for Plant Breeding and Cultivar Recommendations.

[6] Olivoto T., Lúcio A.D., Silva J.A., Sari B.G., Diel M.I. (2019). Mean performance and stability in multi-environment trials II: Selection based on multiple traits. Agron. J..

[7] Yan W., Kang M.S., Ma B., Woods S., Cornelius P.L. (2007). GGE biplot vs. AMMI analysis of genotype-by-environment data. Crop Sci..

[8] Ishwarya M.C., Swapnil Rout S., Singh D., Panda K.K., Imam Z., Penaganti J., Rahimi M., Kumar P., Naidu T.R., Sankar N.V.M. (2025). Yield stability of finger millet genotypes assessed by AMMI and GGE biplot analysis across diverse environments. Sci. Rep..

[9] Singhal T., Satyavathi C.T., Singh S.P., Sankar M., Malik M., Thribhuvan R., Yadav S., Bharadwaj C. (2024). Elucidating genotype× environment interactions for grain iron and zinc content in a subset of pearl millet (*Pennisetum glaucum*) recombinant inbred lines. Crop Pasture Sci..

[10] Wodebo K.Y., Tolemariam T., Demeke S., Garedew W., Tesfaye T., Zeleke M., Gemiyu D., Bedeke W., Wamatu J., Sharma M. (2023). AMMI and GGE Biplot Analyses for Mega-Environment Identification and Selection of Some High-Yielding Oat (*Avena sativa* L.) Genotypes for Multiple Environments. Plants.

[11] Demelash H. (2024). Genotype by environment interaction, AMMI, GGE biplot, and mega environment analysis of elite *Sorghum bicolor* (L.) Moench genotypes in humid lowland areas of Ethiopia. Heliyon.

[12] De Assis R.L., De Freitas R.S., Mason S.C. (2018). Pearl millet production practices in Brazil: A review. Exp. Agric..

[13] Davis A., Dale N., Ferreira F. (2003). Pearl millet as an alternative feed ingredient in broiler diets. J. Appl. Poultry Res..

[14] Li H., Huang Y. (2017). Expression of brown-midrib in a spontaneous sorghum mutant is linked to a 5′-UTR deletion in lignin biosynthesis gene *SbCAD2*. Sci. Rep..

[15] Gupta S.K., Govintharaj P. (2023). Inheritance and allelism of brown midrib trait introgressed in agronomically promising backgrounds in pearl millet (*Pennisetum glaucum* (L.) R. Br.). Czech J. Genet. Plant Breed..

[16] Shashikumara P., Harikrishna H., Jain N., Sinha N., Chauhan D., Phuke R.M., Ambati D., Singh J.B., Prasad S.V.S., Singh G.P. (2020). Genetic variability and genotype × environment interaction for grain yield of wheat (*Triticum aestivum*) backcross inbred lines population under different moisture regimes. Indian. J. Agric. Sci..

[17] Taleghani D., Rajabi A., Saremirad A., Fasahat P. (2023). Stability analysis and selection of sugar beet (*Beta vulgaris* L.) genotypes using AMMI, BLUP, GGE biplot and MTSI. Sci. Rep..

[18] Khandelwal V., Patel R., Choudhary K.B., Pawar S.B., Patel M.S., Iyanar K., Mungra K.D., Kumar S., Satyavathi C.T. (2024). Stability analysis and identification of superior hybrids in pearl millet [*Pennisetum glaucum* (L.) R. Br.] using the multi trait stability index. Plants.

[19] Madhusudhana R., Umakanth A.V., Kaul S., Rana B.S. (2003). Stability analysis for grain yield in rabi sorghum [*Sorghum bicolor* (L.) Moench.]. Indian J. Genet. Plant Breed..

[20] Cherney D.J.R., Patterson J.A., Johnson K.D. (1990). Digestibility and feeding value of pearl millet as influenced by the brown-midrib, low-lignin trait. J. Anim. Sci..

[21] Hanna W.W., Monson W.G., Gaines T.P. (1981). IVDMD, Total Sugars, and Lignin Measurements on Normal and Brown Midrib (bmr) Sorghums at Various Stages of Development 1. Agron. J..

[22] Sattler S.E., Saballos A., Xin Z., Funnell-Harris D.L., Vermerris W., Pedersen J.F. (2014). Characterization of novel sorghum brown midrib mutants from an EMS-mutagenized population. G3 Genes Genomes Genet..

[23] Oliver A.L., Pedersen J.F., Grant R.J., Klopfenstein T.J. (2005). Comparative effects of the sorghum bmr-6 and bmr-12 genes: I. Forage sorghum yield and quality. Crop Sci..

[24] Gangashetty P., Yadav C.B., Riyazaddin M., Vermula A., Asungre P.A., Angarawai I., Mur L.A., Yadav R.S. (2023). Genotype-by-environment interactions for starch, mineral, and agronomic traits in pearl millet hybrids evaluated across five locations in West Africa. Front. Plant Sci..

[25] Mondal B., Singh A., Yadav A., Tomar R.S.S., Vinod Singh G.P., Prabhu K.V. (2017). QTL mapping for early ground cover in wheat (*Triticum aestivum* L.) under drought stress. Curr. Sci..

[26] Gauch H.G. (2013). A simple protocol for AMMI analysis of yield trials. Crop Sci..

[27] Boratkar M.V., Bhivgade S.W., Manza G.R., Shivade H.A., Pareek S.T., Atkari D.G. (2023). AMMI and GGE biplot analysis of genotype by environment interaction and yield stability in pearl millet [*Pennisetum glaucum* (L.) R. Br.]. Int. J. Agric. Sci..

[28] Narasimhulu R., Veeraraghavaiah R., Reddy B.S., Satyavathi C.T., Ajay B.C., Reddy P.S. (2023). Yield stability analysis of pearl millet genotypes in arid region of India using AMMI and GGE biplot. J. Environ. Biol..

[29] Patel R., Parmar D.J., Kumar S., Patel D.A., Memon J., Patel M.B., Patel J.K. (2023). Dissection of genotype × environment interaction for green cob yield using AMMI and GGE biplot with MTSI for selection of elite genotype of sweet corn (*Zea mays* conva. *Saccharata* var. *rugosa*). Indian J. Genet. Plant Breed..

[30] Olivoto T., Nardino M., Meira D., Meier C., Follmann D.N., de Souza V.Q., Konflanz V.A., Baretta D. (2021). Multi-trait selection for mean performance and stability in maize. Agron. J..

[31] Behera P.P., Singode A., Bhat B.V., Ronda V., Borah N., Verma H., Gogoi L.R., Borah J.L., Majhi P.K., Saharia N. (2024). Genetic gains in forage sorghum for adaptive traits for non-conventional area through multi-trait-based stability selection methods. Front. Plant Sci..

[32] Van Soest P.J. (1963). Use of Detergents in the Analysis of Fibrous Feeds. II. A Rapid Method for the Determination of Fiber and Lignin. J. Assoc. Off. Anal. Chem..

