# Supplementary material for: Multi-Environment Evaluation and Stability Analysis for the Selection of Elite Pearl Millet Genotypes with Better Fodder Yield and Quality Component Traits
Source: Plants (Basel). 2026 Mar 27;15(7):1034. doi: 10.3390/plants15071034 (PMC13075082; doi:10.3390/plants15071034)
Supplement: Supplementary file 1 [file plants-15-01034-s001.zip › plants-4139253-supplementary.docx]

| **SL.No.** | **G. Name** | **G. No** | **Pedigree** | **Genetic constitution** | **Origin** | **Selection criteria/trait targeted** |
| --- | --- | --- | --- | --- | --- | --- |
| 1 | ICMbmr 2401 | IGPM-1 | ICMV 155 bmr S1-4-2-1-3-4-3-B-P5 | Inbred | ICRISAT, India | Low lignin content |
| 2 | ICMbmr 2402 | IGPM-2 | ICMV 155 bmr S1-4-2-1-3-4-2-B-B-P1 | Inbred | ICRISAT, India | Low lignin content |
| 3 | ICMbmr 2403 | IGPM-3 | WRajPop bmr S1-19-7-3-3-2-2-1-B-B-B-B-P5 | Inbred | ICRISAT, India | Low lignin content |
| 4 | ICMbmr 2404 | IGPM-4 | WRajPop bmr S1-19-7-3-3-2-2-1-B-B-B-B-P6 | Inbred | ICRISAT, India | Low lignin content |
| 5 | ICFPM 02 | IGPM-6 | IP 3471 S1-8-1-1 | Inbred | ICRISAT, India | High tillering, longer leaf length, |
| 6 | ICFPM 03 | IGPM-7 | IP 12128 SS-4-1 | Inbred | ICRISAT, India | High tillering, longer leaf length, |
| 7 | ICFPM 04 | IGPM-8 | IP 12128 SS-4-2 | Inbred | ICRISAT, India | High tillering, longer leaf length, |
| 8 | ICFPM 05 | IGPM-9 | IP 13016 S1-1-5-6 | Inbred | ICRISAT, India | Higher leaf to stem ration |
| 9 | ICFPM 06 | IGPM-10 | IP 24666 S1-3-4-4 | Inbred | ICRISAT, India | Higher leaf to stem ration |
| 10 | ICFP 04 | IGPM-11 | ICMB 10999 | Inbred | ICRISAT, India | High digestibility, |
| 11 | ICFP 05 | IGPM-12 | ICMB 08999 | Inbred | ICRISAT, India | High digestibility, |
| 12 | ICFP 06 | IGPM-13 | ICMB 12111 | Inbred | ICRISAT, India | High digestibility, |
| 13 | ICFPM 07 | IGPM-14 | ICMR 100363 | Inbred | ICRISAT, India | High digestibility, |
| 14 | ICMFV 2308 | IGPM-15 | Forage variety bred at ICRISAT by sib mating of selected progenies derived from land race IP 15536 from Burkina Faso | Open Pollinated Variety (OPV) | ICRISAT, India | Late flowering, high biomass |
| 15 | ICMFV 2401 | IGPM-16 | Forage variety bred at ICRISAT by sib mating of selected S_2_ progenies derived from land race IP 17435 from Central African Republic | Open Pollinated Variety (OPV) | ICRISAT, India | Late flowering, high biomass |
| 16 | ICBbmr 09 | IGPM-17 | WRajPbmrS1-19-7-3-3-2-2-1-B-B | Inbred | ICRISAT, India | Low lignin content |
| 17 | IGPM100 | IGPM-18 | Forage line developed by crossing between selected S2 progenies of IP20345 and IP 6796 | Open Pollinated Variety (OPV) | India | Delayed flowering, higher leaf to stem ratio, |
| 18 | IGBV97 | IGPM-19 | Forage line developed through mass selection from forage population developed using germplasm line PE5903 | Open Pollinated Variety (OPV) | India | High biomass, better regrowth ability |
| 19 | IGBV 9 | IGPM-21 | Forage line developed through mass selection from forage population developed using germplasm line IP11336 | Open Pollinated Variety (OPV) | India | High biomass, better regrowth ability |
| 20 | Baif bajra 1 | IGPM-22 | Selection from base population of Giant Bajra | Open Pollinated Variety (OPV) | India | Released variety, high biomass, better regrowth ability |
| 21 | Giant bajra | IGPM-23 | Derivative of the cross between Australian Bajra x Local Bajra (from Dhule district) | Open Pollinated Variety (OPV) | India | Released variety, high biomass, better regrowth ability |
| 22 | IGBV 128 | IGPM-24 | IP20440-S1-S2-S3 selection from crossing S3 generation intercross | Open Pollinated Variety (OPV) | India | High biomass, better regrowth ability |
| 23 | IGPM 1134 | IGPM-25 | IP 18389-S1---S7-B | Inbred | India | Late flowering, high tillering, |
| 24 | IGPM 1035 | IGPM-26 | IP 7633-S1----S7-B | Inbred | India | Late flowering, high tillering, |
| 25 | IGPM1106 | IGPM-27 | PT 732B-P2- S1----S7-B | Inbred | India | Late flowering, high tillering, |
| 26 | IGPM1109 | IGPM-28 | IP 8002- S1----S7-B | Inbred | India | Late flowering, high tillering, |
